# Multidisciplinary management of acute type A aortic dissection presenting with atypical symptoms, abdominal hemorrhage, and Rh-negative blood type: a case report

**DOI:** 10.3389/fcvm.2026.1833386

**Published:** 2026-08-26

**Authors:** Yuning Xi, Meng Yu, Yuhang Lv, Liang Dong

**Affiliations:** 1 Department of Gastrointestinal and Colorectal Surgery, Taizhou Central Hospital, Taizhou, China; 2 Department of Intensive Care Medicine (ICU), Taizhou Central Hospital, Taizhou, China

**Keywords:** abdominal hemorrhage, ischemia-reperfusion injury, multidisciplinary team, Rh-negative blood, type A aortic dissection

## Abstract

**Background:**

Acute type A aortic dissection (TAAD) is a catastrophic cardiovascular emergency with high mortality. Patients presenting with atypical gastrointestinal symptoms, such as abdominal pain and diarrhea, are particularly prone to misdiagnosis. Visceral branch vessel injury caused by the dissection can directly lead to abdominal hemorrhage, a rare but fatal complication.

**Case summary:**

We report a case of a 66-year-old man who presented with a one-day history of abdominal pain and diarrhea, followed by chest tightness and shortness of breath for 10 hours. Computed tomography angiography (CTA) revealed a DeBakey type I TAAD involving the celiac trunk and superior mesenteric artery, with suspected active abdominal hemorrhage. The patient had a rare Rh-negative B blood type. Emergency “Sun's procedure” was performed. Postoperatively, the pre-existing abdominal hemorrhage was exacerbated by coagulopathy and surgical stress, leading to hemorrhagic shock. Following multidisciplinary discussion, given the diffuse nature of the bleeding, a stepwise hemostatic strategy of “interventional embolization first, surgical exploration as backup” was adopted. The patient subsequently developed liver dysfunction and diarrhea, which may be attributed to visceral ischemia-reperfusion injury. He ultimately recovered after comprehensive management.

**Conclusion:**

Unexplained shock with acute abdominal pain, especially in hypertensive patients, should raise suspicion for TAAD and prompt early aortic CTA. TAAD-related abdominal hemorrhage may be recognized as a manifestation of systemic vascular injury. Management may benefit from an understanding of the multi-factor cascading pathophysiological model and could follow a stepwise hemostatic approach. In the context of massive hemorrhage with a rare blood type, a rapid blood supply system is essential. Meticulous perioperative monitoring and efficient multidisciplinary collaboration are cornerstones of successful resuscitation in such complex cases.

## Introduction

Acute type A aortic dissection (TAAD) remains one of the most lethal cardiovascular emergencies, with in-hospital mortality rates as high as 57% despite surgical advances (1). Single-center data from China report a postoperative in-hospital mortality of 10.5% (2), which differs from the 18.1% reported by the Sino-RAD registry (3), likely reflecting variations in disease severity among enrolled patients.

The classic presentation of TAAD involves sudden, severe chest or back pain. However, approximately 6%–10% of patients present with atypical gastrointestinal symptoms such as abdominal pain and diarrhea (4, 5). These patients experience diagnostic delays and significantly higher mortality compared to those with typical symptoms (28% vs. 10%, *p* = 0.02) (6). In the Chinese population, the incidence of abdominal pain is approximately 12.0%, with back pain remaining the predominant symptom, differing from Western cohorts (3). When the dissection involves the visceral branches of the abdominal aorta, it not only causes atypical symptoms but can also directly disrupt vascular integrity, leading to abdominal hemorrhage. The 2024 ESC Guidelines for the management of peripheral arterial and aortic diseases emphasize that patients with acute TAAD presenting with malperfusion (mesenteric, renal, lower limb, or cerebral) require emergency surgery to improve outcomes (7). This type of bleeding should be considered a clinical manifestation of systemic vascular injury during the acute phase of TAAD. Herein, we report a case of TAAD presenting with abdominal pain and diarrhea, complicated by pre-existing dissection-related abdominal hemorrhage, R-negative blood type, and postoperative hemorrhagic exacerbation, which was successfully managed through multidisciplinary collaboration.

## Case presentation

A 66-year-old man presented to our hospital on November 4, 2025, with a one-day history of abdominal pain and diarrhea, followed by chest tightness and shortness of breath for over 10 hours. The abdominal pain was periumbilical, colicky, and began after a fatty meal. He reported increased stool frequency (7–8 times/day) with yellowish, pasty stools, without fever or hematochezia. Ten hours prior, he was evaluated at a local hospital, where he was found to be hypotensive (70/40 mmHg). Fluid resuscitation and vasopressors provided minimal improvement. During a thoracoabdominal CTA, he acutely developed chest tightness, shortness of breath, and desaturation, necessitating emergent endotracheal intubation.

### Imaging findings

**Figure 1:**
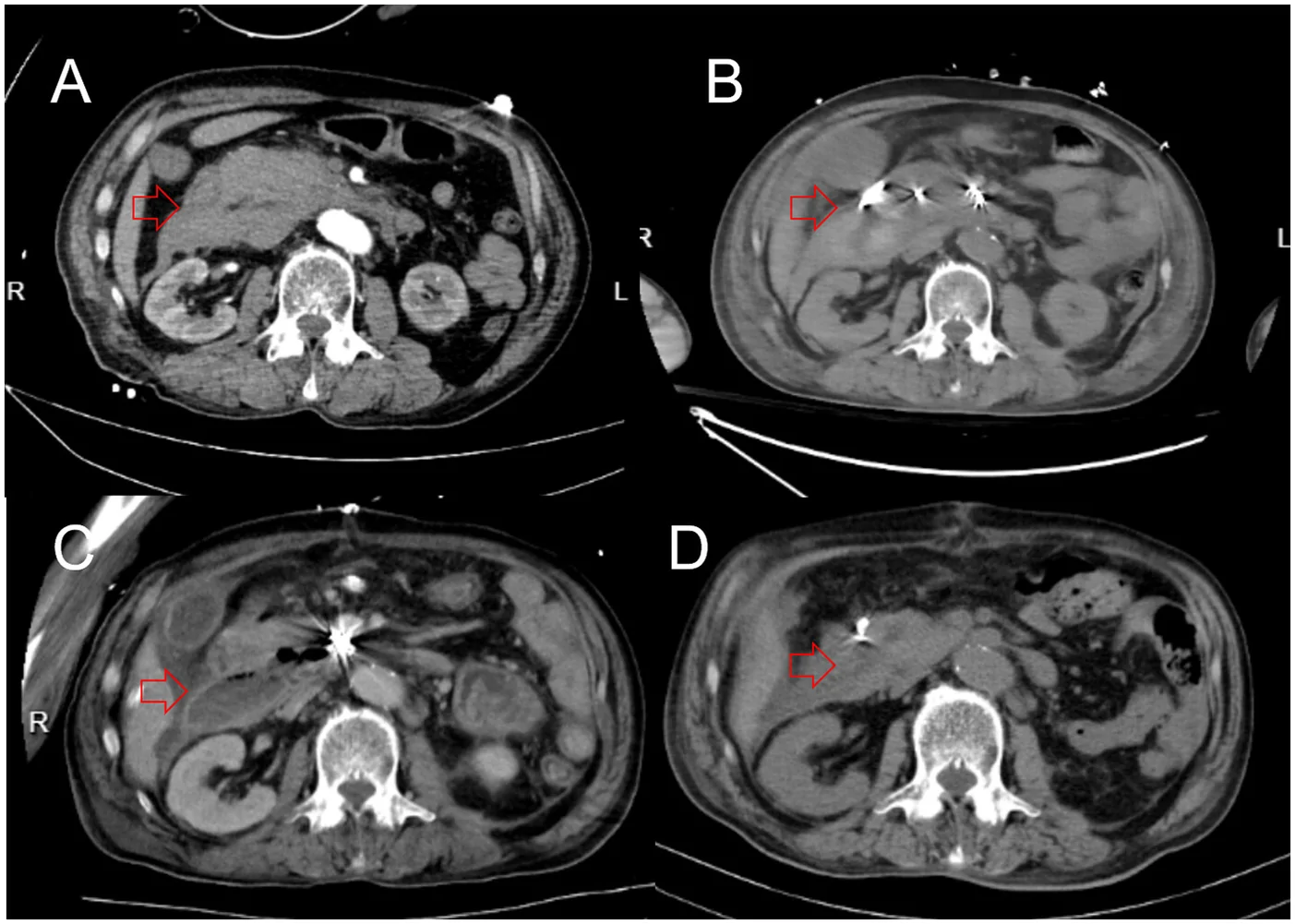
Serial abdominal CT findings. **(A)** Preoperative CTA showing a hyperdense area with nodular contrast extravasation (arrow) in the abdominal cavity, indicating active hemorrhage. **(B)** CT after interventional embolization and before exploratory laparotomy, demonstrating enlargement of the abdominal hematoma. **(C)** CT after exploratory laparotomy, showing evacuation of the hematoma and packing. **(D)** CT before discharge, revealing significant resolution of the hematoma.

CTA revealed a DeBakey type I aortic dissection involving the right common carotid and left subclavian arteries. The celiac trunk, superior mesenteric artery, and right renal artery originated from the false lumen, with severe compression of the true lumen. A hyperdense area with nodular contrast extravasation was observed in the abdominal cavity, indicating active hemorrhage (Figure 1A), likely due to direct injury to visceral branches by the dissection.

### Medical history

The patient had a 30-year smoking history, with a consumption of 1–2 packs per day, and had not quit smoking. He had a history of alcohol consumption (specific quantity unknown) and had not abstained. He had a known history of hypertension for over 10 years but did not monitor his blood pressure regularly and was not on any antihypertensive medication. He denied any family history of aortic dissection, connective tissue disorders, or sudden death, and denied any history of diabetes mellitus or coronary artery disease.

### Physical examination on arrival

On arrival at our institution, the patient was intubated and mechanically ventilated, under sedation. Vital signs were as follows: temperature 36.9°C, pulse 90 beats/min, respiratory rate 27 breaths/min, and blood pressure 94/52 mmHg. Cardiac auscultation revealed no significant murmurs. The abdomen was soft; however, due to the patient's sedated state, tenderness and rebound tenderness could not be reliably assessed. Neurological examination showed spontaneous movement of all extremities.

Upon transfer to our institution, the patient underwent emergency “Sun's procedure” under general anesthesia with cardiopulmonary bypass (CPB), consisting of ascending aortic replacement, aortic valve replacement (Bentall procedure), total arch replacement, and frozen elephant trunk implantation. The operative time was 10.5 hours, with an aortic cross-clamp time of 164 minutes and a CPB time of 301 minutes. Estimated intraoperative blood loss was 1000 mL. He received 2 units of leukoreduced red blood cells (RBCs), 1000 mL of fresh frozen plasma, 20 units of platelets, and 750 mL of autologous blood transfusion.

### Postoperative course

Later that same day, the patient developed progressive hypotension and a sharp decline in hemoglobin concentration. Bedside ultrasound revealed a significant increase in the size of the abdominal hematoma compared to the immediate postoperative scan. A multidisciplinary discussion concluded that the pre-existing dissection-related abdominal hemorrhage was exacerbated by postoperative coagulopathy (consumptive coagulopathy, heparinization) and surgical stress, resulting in hemorrhagic shock. Given the patient's rare Rh-negative B blood type, an emergency blood product mobilization was initiated. To achieve life-saving resuscitation, after obtaining informed consent regarding the risks and benefits, Rh-positive RBCs and plasma were transfused. Subsequently, digital subtraction angiography (DSA) was performed, and embolization of branches of the gastroduodenal and superior mesenteric arteries was successfully carried out using coils. His blood pressure stabilized temporarily.

However, on postoperative day 8 (November 12), the patient again developed progressive abdominal distension and a decreasing hemoglobin level. Repeat CT scan showed an enlarging abdominal hematoma (Figure 1B). Following another multidisciplinary discussion, persistent bleeding despite embolization was suspected, and exploratory laparotomy was performed on November 17 (postoperative day 13). Intraoperatively, a large retroperitoneal hematoma was evacuated. No single point of active bleeding was identified, consistent with diffuse oozing from TAAD-related vascular injury. The abdomen was packed, and hemostatic sutures were placed for reinforcement.

**Figure 2:**
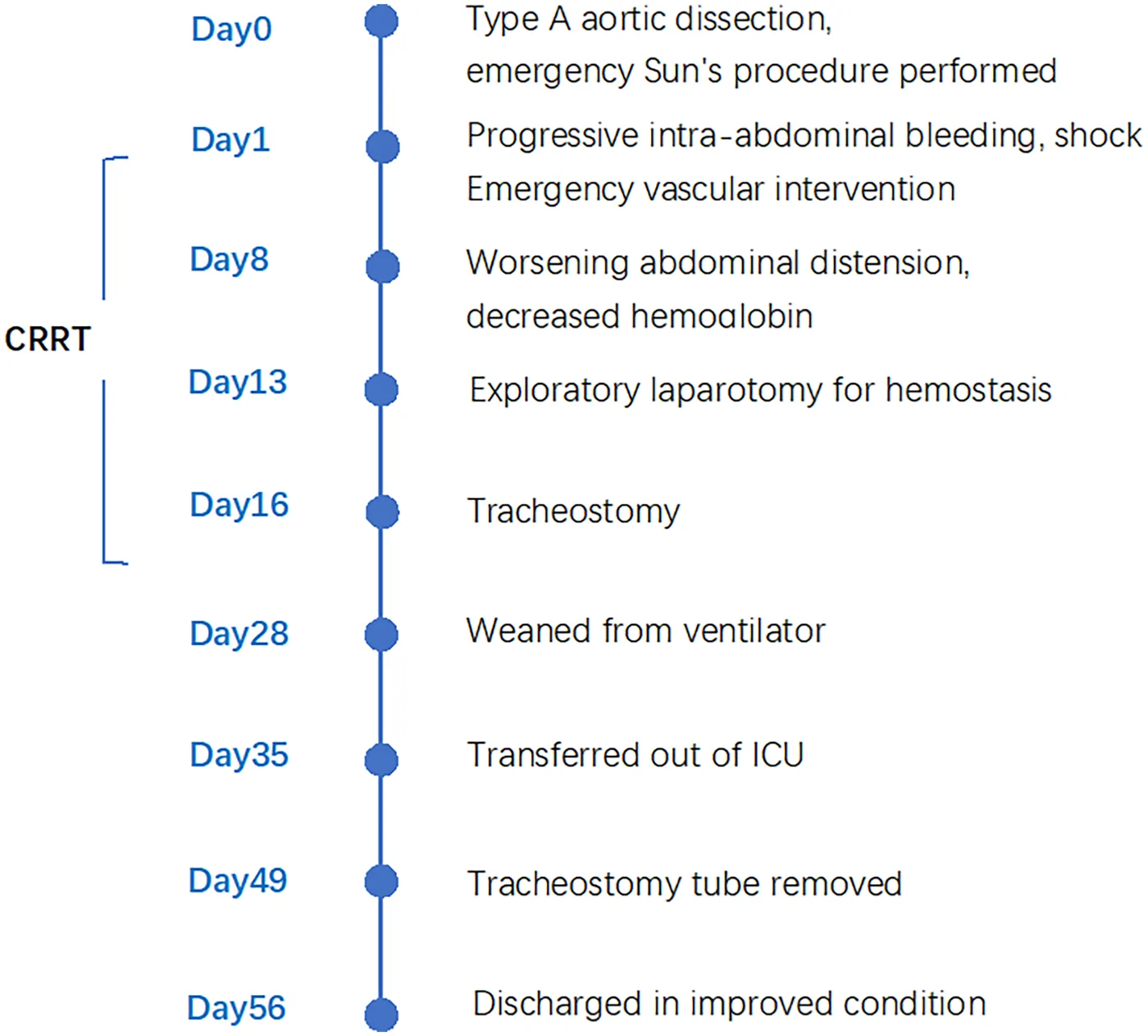
Timeline of the patient's clinical course. Key events include initial presentation, CTA diagnosis, emergency Sun's procedure, postoperative hemorrhagic shock with DSA embolization, recurrent bleeding on day 8 leading to exploratory laparotomy on day 13, CRRT from day 2 to day 14, gradual recovery, and discharge on postoperative day 56.

Postoperatively, the patient's total bilirubin peaked at 133 μmol/L (predominantly direct bilirubin), attributed to a combination of Rh-incompatible transfusion, hepatic hypoperfusion from hemorrhagic shock, and ischemia-reperfusion injury. He also developed severe diarrhea. Given the preoperative CTA findings of superior mesenteric artery involvement and intraoperative observation of compromised intestinal perfusion, the diarrhea was primarily attributed to TAAD-related intestinal ischemia and reperfusion-induced edema. Although testing for Clostridioides difficile toxin was negative, secondary infection due to impaired gut barrier function could not be entirely excluded. The patient received continuous renal replacement therapy (CRRT) from November 6 to November 20 (intermittent sessions) due to acute kidney injury with worsening renal function, rising myoglobin, and urine output <0.5 mL/kg/h for 12 hours. With comprehensive supportive care, including efforts to optimize perfusion, and oral vancomycin, the diarrhea gradually improved. The patient was successfully weaned from mechanical ventilation and transferred to the general ward. He was discharged home on postoperative day 56 with significant recovery. Total blood product transfusion during the entire hospitalization: RBCs 35 units, plasma 4750 mL, platelets 40 units, cryoprecipitate 20 units, and autologous blood 750 mL. Intraoperative transfusion details are summarized in the revised manuscript. Figure 2 summarizes the patient's clinical course in a timeline.

## Discussion

### Abdominal hemorrhage as a manifestation of systemic vascular injury in TAAD

This patient presented with abdominal pain and diarrhea, lacking classic chest or back pain. Preoperative CTA demonstrated involvement of the celiac trunk and superior mesenteric artery with active abdominal hemorrhage. Such atypical presentations significantly increase the risk of diagnostic delay. The mortality rate for aortic dissection patients presenting primarily with abdominal pain is significantly higher than for those with classic symptoms (28% vs. 10%, *p* = 0.02) (1, 6), with up to one-third initially misdiagnosed with gastrointestinal disease (5); each hour of diagnostic delay increases mortality risk significantly in untreated TAAD, with mortality rates estimated at 1–2% per hour during the first 48 hours (8, 9). This patient exhibited the “lethal triad” identified by the International Registry of Acute Aortic Dissection (IRAD)—hypotension/shock, absence of chest pain, and branch vessel involvement—all significant predictors of mortality in type A dissection (OR 23.8, 3.5, and 2.9, respectively; all *p* < 0.05) (1). The 2024 ESC Guidelines emphasize the use of a multiparametric algorithm combining the aortic dissection detection risk score (ADD-RS) and D-dimer for rapid diagnosis in patients with suspected acute aortic syndrome, followed by early aortic CTA (7). This underscores the critical need for clinicians to consider TAAD in the differential diagnosis for any patient with acute abdominal pain accompanied by unexplained shock, particularly those with a history of hypertension, and to perform early aortic CTA (5, 8).

Although mesenteric malperfusion complicating TAAD is rare (3.7%), it carries a prohibitive in-hospital mortality rate, even with surgical repair (42%) (1). The 2024 ESC Guidelines recommend emergency surgery for patients with acute TAAD presenting with malperfusion, including mesenteric involvement (7). In our patient, preoperative CTA findings of celiac trunk and superior mesenteric artery involvement with active abdominal hemorrhage were a direct consequence of the widespread disruption of vascular integrity caused by the DeBakey type I TAAD. Therefore, such hemorrhage should be recognized as an integral component of systemic vascular injury during the acute phase of TAAD. In patients with TAAD involving the abdominal arteries, a high index of suspicion for this fatal complication is warranted at initial presentation.

### Multi-factor cascading pathophysiological model for postoperative hemorrhagic exacerbation

The life-threatening abdominal hemorrhage that occurred postoperatively in this case resulted from a cascade of amplifying factors. First, DeBakey type I dissection itself is an independent risk factor for major postoperative bleeding (OR = 2.652) (10). Acute aortic dissection can trigger consumptive coagulopathy, leaving patients with impaired hemostatic function preoperatively (11). In this case, the visceral arteries (celiac trunk, superior mesenteric artery) were perfused by the false lumen, rendering their structural integrity fundamentally compromised and providing the anatomical substrate for bleeding (10, 12). Second, the systemic heparinization (300–400 U/kg) required for cardiac surgery and potential postoperative heparin rebound further destabilized an already fragile coagulation balance. Third, prolonged CPB (301 minutes) and deep hypothermic circulatory arrest can decrease platelet count by 30%–50%, impair platelet function, and activate the fibrinolytic system (12); intraoperative hypotension from blood loss or reperfusion exacerbated ischemia-reperfusion injury to the abdominal viscera. The convergence of these three layers of factors transformed the pre-existing unstable vascular bed into a diffuse, uncontrollable hemorrhagic state postoperatively. Re-exploration for bleeding within 24 hours post-surgery is an independent risk factor for both in-hospital and long-term mortality (in-hospital death OR = 3.109; long-term death HR = 3.039), with in-hospital mortality rates for major bleeding reaching 30.3% (10). The 2024 ESC Guidelines recommend immediate surgical intervention for patients with acute TAAD presenting with malperfusion (7). Our patient, under this high-risk scenario, ultimately achieved hemostasis through a multidisciplinary, stepwise approach.

### A stepwise hemostatic strategy based on pathophysiological characteristics

Guided by the pathological characteristic of diffuse, non-focal bleeding, we implemented a stepwise hemostatic strategy. Initially, based on clearly identified contrast extravasation sites on preoperative CTA and intraoperative DSA, superselective embolization was performed to address accessible arterial bleeding branches minimally invasively. When bleeding persisted after intervention, suggesting multiple vessel oozing, venous bleeding, or widespread tissue oozing, exploratory laparotomy was decisively undertaken. Intraoperatively, a large retroperitoneal hematoma was evacuated, and no single point of active bleeding was identified, consistent with diffuse oozing from TAAD-related vascular injury. Abdominal packing and reinforcing hemostatic sutures were applied. Delaying intervention for active postoperative bleeding significantly increases mortality (10). The 2024 ESC Guidelines recommend that patients with acute TAAD be evaluated and treated in high-volume aortic centres with multidisciplinary teams to improve survival, provided transfer does not significantly delay surgery (7). Given the patient's DeBakey type I dissection (OR = 2.652 for major bleeding) (10), acute consumptive coagulopathy (11), and further exacerbation of coagulopathy by prolonged CPB and deep hypothermic circulatory arrest (11), the bleeding risk was exceptionally high. In this context, the “intervention first, surgery as backup” approach represents a clinical pathway informed by the recognized complexity of post-TAAD bleeding. Although re-exploration for bleeding increases mortality risk more than threefold (10), timely reintervention for severe postoperative complications can offer patients a chance for long-term survival (13). This case suggests that a stepwise hemostatic strategy may be clinically applicable for managing diffuse abdominal bleeding after TAAD repair.

### Massive transfusion strategy and evidence-based decision-making in a critically ill patient with a rare blood type

This patient's Rh-negative B blood type posed a critical challenge during acute, life-threatening abdominal hemorrhage due to the scarcity of compatible blood products. In acute hemorrhagic shock, restoring blood volume and oxygen-carrying capacity is the highest priority. In emergency situations where type-specific blood is unavailable, transfusion of ABO-compatible Rh-positive blood is an accepted life-saving measure (15). Although this may sensitize Rh-negative recipients and lead to anti-D antibody formation, the immediate risk of death must be weighed against the potential for future complications; clinical decision-making should prioritize the immediate survival benefit (15). Current clinical practice guidelines explicitly state that in life-threatening bleeding emergencies where Rh-negative products are unavailable, Rh-positive products should be used regardless of patient age or sex, as the immediate survival benefit outweighs hypothetical future risks (15). Our patient, an elderly male with no future childbearing potential, received Rh-positive RBCs after thorough risk disclosure and informed consent, buying time for definitive subsequent interventions. This decision aligns with the “life-first” principle and is supported by evidence-based guidelines. The postoperative hyperbilirubinemia observed in this patient was likely multifactorial, related not only to ischemia-reperfusion injury but also to massive transfusion (14, 16), warranting close monitoring in subsequent management. Anti-D antibody testing was not performed after discharge, which we acknowledge as a limitation.

### The postoperative complication cascade: a continuation of ischemia-reperfusion injury

The postoperative hyperbilirubinemia and severe diarrhea in this case should be understood within the framework of the underlying TAAD pathology. Preoperative CTA showed the celiac trunk and superior mesenteric artery arising from the false lumen, a high-risk anatomical pattern for static obstruction (12). The false lumen, with its exposed medial tissue and prothrombotic state, is prone to thrombosis (12), which explains how mesenteric ischemia may have occurred postoperatively during true lumen expansion and false lumen compression. Hemorrhagic shock and perioperative hypoperfusion led to hepatic ischemia; subsequent reperfusion generated oxygen free radicals, exacerbating hepatocellular injury and manifesting as cholestasis and hyperbilirubinemia. Concurrently, superior mesenteric artery involvement directly caused intestinal ischemia, and reperfusion injury after resuscitation resulted in bowel wall edema and barrier disruption, clinically presenting as diarrhea. The negative C. difficile toxin assay, combined with imaging and intraoperative findings, supports TAAD-related intestinal ischemia and reperfusion edema as the primary cause of diarrhea. Alternative explanations for diarrhea—including antibiotic-associated diarrhea and enteral feeding intolerance—remain possible, although the negative C. difficile assay and the resolution of symptoms with supportive care support our preferred interpretation. With comprehensive supportive care, including optimizing perfusion, the diarrhea gradually resolved. Literature suggests that gastrointestinal complications occur in 70–80% of patients after TAAD repair, with gut dysbiosis playing a significant role (16). Our patient ultimately recovered through meticulous dynamic monitoring, early recognition of hyperbilirubinemia, and targeted interventions. This underscores that for post-TAAD complications, a coherent intervention strategy based on the ischemia-reperfusion injury mechanism may be crucial for multi-organ support (17).

### Central repair vs. reperfusion-first strategy in TAAD with mesenteric malperfusion

The optimal management strategy for TAAD with concomitant mesenteric malperfusion remains debated. The 2022 ACC/AHA guidelines suggest that either immediate central aortic repair or immediate mesenteric revascularization before aortic repair is reasonable (Class 2a) in patients with clinically significant malperfusion. Recent data by Gao et al. (2024) showed that a reperfusion-first strategy with SMA stenting may be associated with lower mortality compared to central repair first (19.4% vs. 48.6%, *P* = 0.012) in select patients. However, the decision must be individualized. In our case, the patient presented with hemodynamic instability and active intra-abdominal hemorrhage, which we considered to pose a more immediate threat than isolated mesenteric ischemia. We chose central aortic repair first with the expectation that addressing the primary entry tear would decompress the false lumen and improve visceral perfusion. This decision was made after multidisciplinary discussion, balancing the risk of aortic rupture against the potential benefits of upfront mesenteric reperfusion.

### Alternative explanations and proposed mechanisms

We acknowledge that several pathophysiological interpretations in this case remain speculative. The postoperative hyperbilirubinemia may also be explained by drug-induced hepatotoxicity (e.g., from vancomycin or sedatives), transfusion-related iron overload, or hepatic hypoperfusion from shock. While we cannot definitively exclude these, we favor ischemia-reperfusion injury as the primary mechanism given the temporal relationship with hemorrhagic shock and the predominance of direct bilirubin elevation, which is more consistent with cholestatic injury than with drug-induced hepatocellular damage. Similarly, the diarrhea may have been contributed to by antibiotic-associated dysbiosis or enteral feeding intolerance; however, the negative C. difficile assay and the absence of specific anti-infective therapy support intestinal ischemia-reperfusion as the primary etiology. The “multi-factor cascading model” we propose is intended as a conceptual framework to guide clinical reasoning rather than as a proven pathophysiological pathway.

### Limitations

As a single case report, this study has inherent limitations. The findings and management strategies described here may not be generalizable to all patients with TAAD and abdominal hemorrhage, as clinical presentation and anatomical characteristics vary considerably. The stepwise hemostatic approach we describe—interventional embolization followed by surgical exploration—may be most applicable to patients with hemodynamic stability and anatomically accessible bleeding targets. The clinical message from this case is primarily educational: it illustrates a potential approach to a complex scenario, but treatment decisions should continue to be individualized based on patient-specific factors and institutional expertise.

## Conclusion

This case demonstrates the successful multidisciplinary management of a complex TAAD patient presenting with gastrointestinal symptoms, pre-existing dissection-related abdominal hemorrhage, and a rare Rh-negative blood type. The key takeaways are: (1) TAAD-related abdominal hemorrhage may be recognized as a manifestation of systemic vascular injury; (2) management may benefit from a deep understanding of the “multi-factor cascading” pathophysiological model for postoperative hemorrhagic exacerbation, guiding the implementation of a stepwise hemostatic strategy—"interventional embolization first, surgical exploration as backup"—particularly suitable for diffuse bleeding; (3) in the extreme circumstance of massive hemorrhage in a patient with a rare blood type, evidence-based emergency transfusion decisions, including the judicious use of ABO-compatible Rh-positive blood after weighing risks and benefits, may be life-saving. Ultimately, rapid response and seamless collaboration within a multidisciplinary team are the cornerstones that integrate these insights, enabling precise intervention and overcoming such formidable clinical challenges.

## Patient perspective

The patient expressed his gratitude to the entire medical team for saving his life. After discharge, he presented our department with a commemorative banner as a token of appreciation for the care he received. He stated that although the experience was terrifying, he felt supported throughout the treatment process. He now understands the importance of managing his blood pressure and adhering to regular follow-up.

## Data Availability

The original contributions presented in the study are included in the article/Supplementary Material, further inquiries can be directed to the corresponding author.
